# Case Report: Solitary right thigh skin metastasis of bladder cancer misdiagnosed as erysipelas with literature review

**DOI:** 10.3389/fonc.2026.1825362

**Published:** 2026-09-03

**Authors:** Zhaonan Hou, Mingfeng Ji, Fang Peng, Yihao Chen, Zhiyu Liu, Zhihong Dai

**Affiliations:** 1 Department of Urology, The Second Affiliated Hospital of Dalian Medical University, Dalian, China; 2 Department of Pathology, The Second Affiliated Hospital of Dalian Medical University, Dalian, China

**Keywords:** bladder cancer, case report, cutaneous metastasis, immunotherapy, misdiagnosis

## Abstract

**Background:**

Cutaneous metastasis from bladder carcinoma is an uncommon clinical manifestation and may be misdiagnosed as a benign inflammatory dermatosis, such as erysipelas, causing diagnostic delay and missed opportunities for timely systemic treatment.

**Case presentation:**

We report a case of a 70-year-old man with a history of muscle-invasive bladder carcinoma treated with radical cystectomy and bilateral cutaneous ureterostomies, who presented 6 years later with a maculopapular rash on his right thigh. The lesion was initially misdiagnosed and treated as erysipelas for over 2 months without improvement. A subsequent skin biopsy confirmed metastatic urothelial carcinoma, with immunohistochemistry positive for GATA-3, CK7, and CK20. The tumor also exhibited Human epidermal growth factor receptor 2 (HER2) (2+) expression and high Programmed death-ligand 1 (PD-L1) (Combined Positive Score (CPS) = 20). Based on prior intolerance to cisplatin-based chemotherapy and the tumor biomarker profile, combination therapy with disitamab vedotin and tislelizumab was initiated. This regimen led to complete resolution of pain by cycle 6, and achieved complete clinical and radiological remission of the skin metastasis after 18 cycles of treatment. However, the patient later developed grade 3 immune-related adrenal insufficiency, necessitating hormone replacement and permanent discontinuation of immunotherapy.

**Conclusion:**

Persistent inflammatory-appearing skin lesions in patients with a history of bladder cancer should prompt early biopsy, particularly when they are refractory to antibiotic therapy. Molecular profiling may help identify actionable targets and guide systemic treatment. This case highlights the diagnostic challenge of erysipelas-like cutaneous metastasis and suggests the potential value of biomarker-guided HER2-directed antibody–drug conjugate plus Programmed cell death protein 1 (PD-1) inhibitor therapy in selected patients with metastatic urothelial carcinoma, while emphasizing the need for careful monitoring of immune-related toxicity.

## 1 Introduction

Bladder cancer is one of the most common urological malignancies worldwide, with urothelial carcinoma accounting for the majority of cases (1). For muscle-invasive bladder cancer (MIBC), radical cystectomy with pelvic lymph node dissection and urinary diversion remains a key component of curative-intent treatment (2). However, the treatment landscape for urothelial carcinoma has shifted substantially in recent years, particularly with the emergence of antibody-drug conjugate (ADC) plus immune checkpoint inhibitor (ICI) strategies. In cisplatin-ineligible or cisplatin-declining MIBC, the KEYNOTE-905/EV-303 trial showed that perioperative enfortumab vedotin plus pembrolizumab combined with surgery improved event-free and overall survival compared with surgery alone (3). In locally advanced or metastatic urothelial carcinoma, EV-302/KEYNOTE-A39 further demonstrated superior outcomes with enfortumab vedotin plus pembrolizumab compared with platinum-based chemotherapy (4). Nevertheless, even after definitive local treatment, a substantial proportion of patients experience disease recurrence or develop distant metastases. The most common metastatic sites are regional lymph nodes, bones, lungs, and liver (5, 6).

In comparison, cutaneous metastasis from bladder cancer is an uncommon clinical manifestation and has mostly been described in isolated case reports or small case series (7). Although it is often associated with advanced systemic disease and poor clinical outcomes, survival may vary according to disease burden, metastatic pattern, and response to systemic therapy (8). Clinically, these lesions may mimic benign dermatological conditions such as cellulitis or erysipelas, often resulting in delayed diagnosis and missed opportunities for timely biopsy and systemic treatment (8, 9).

We report a 70-year-old man with recurrent urothelial carcinoma and no significant family history of malignancy, who developed an isolated erysipelas-like cutaneous metastasis on the right thigh 6 years after radical cystectomy, without evidence of visceral metastasis. The lesion was initially misdiagnosed as erysipelas and remained refractory to antibiotic therapy for over 2 months. This report summarizes the clinicopathological characteristics of this case and highlights the diagnostic challenge of inflammatory-appearing cutaneous metastasis. More importantly, the complete clinical and radiological remission achieved after disitamab vedotin plus tislelizumab supports the potential of biomarker-guided HER2-directed ADC plus PD-1 inhibitor therapy in appropriately selected patients with metastatic urothelial carcinoma, while also highlighting the need for careful monitoring of treatment-related immune toxicity.

## 2 Case presentation

In June 2018, following a transurethral resection of bladder tumor (TURBT)-based diagnosis of muscle-invasive bladder cancer, the patient underwent laparoscopic radical cystectomy with bilateral pelvic lymph node dissection and bilateral cutaneous ureterostomies. The final pathological stage was pT2N0M0, and no adjuvant therapy was administered. Thereafter, clinical follow-up was scheduled every 3 months during the first 2 years, every 6 months during years 3–5, and annually thereafter. Contrast-enhanced CT of the chest and abdomen was performed at baseline and annually, or earlier when clinically indicated. Before the onset of overt urethral bleeding in June 2023, the patient recalled mild perineal discomfort and intermittent urethral discharge, but these symptoms were nonspecific. A total urethrectomy was performed, and pathology confirmed local recurrence. He subsequently received three cycles of cisplatin-based chemotherapy but discontinued treatment because of intolerance.

**Figure 1:**
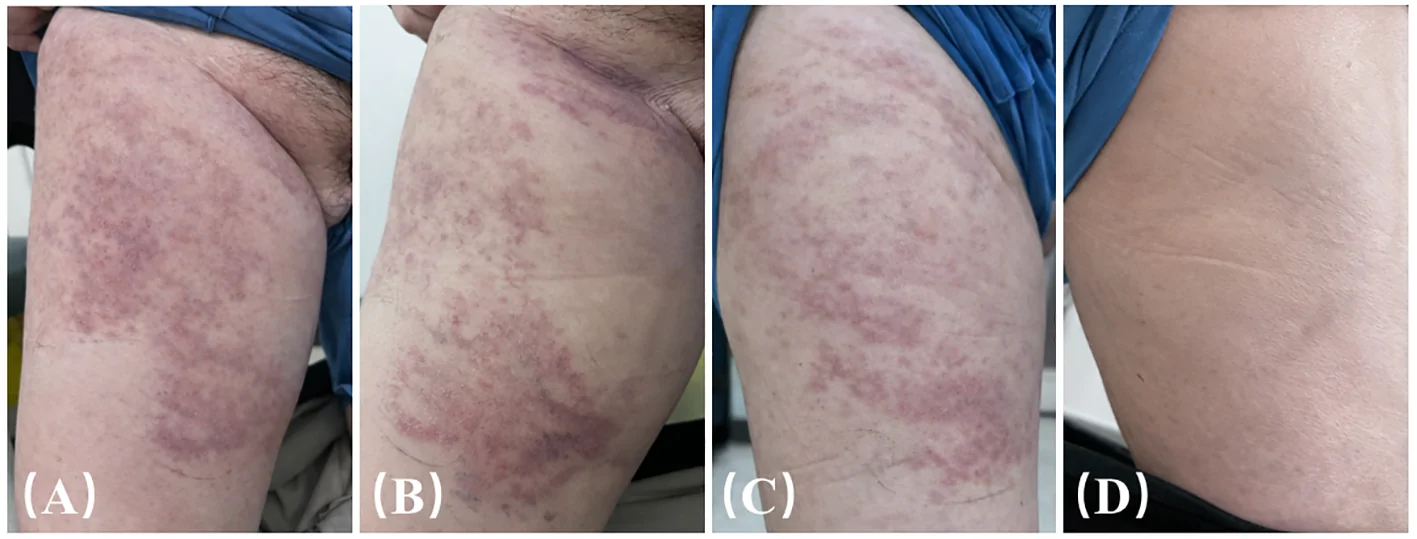
Clinical appearance of the lesion site. **(A)** Anterior view. **(B)** Medial view. **(C)** Lateral view. **(D)** Posterior view.

**Figure 2:**
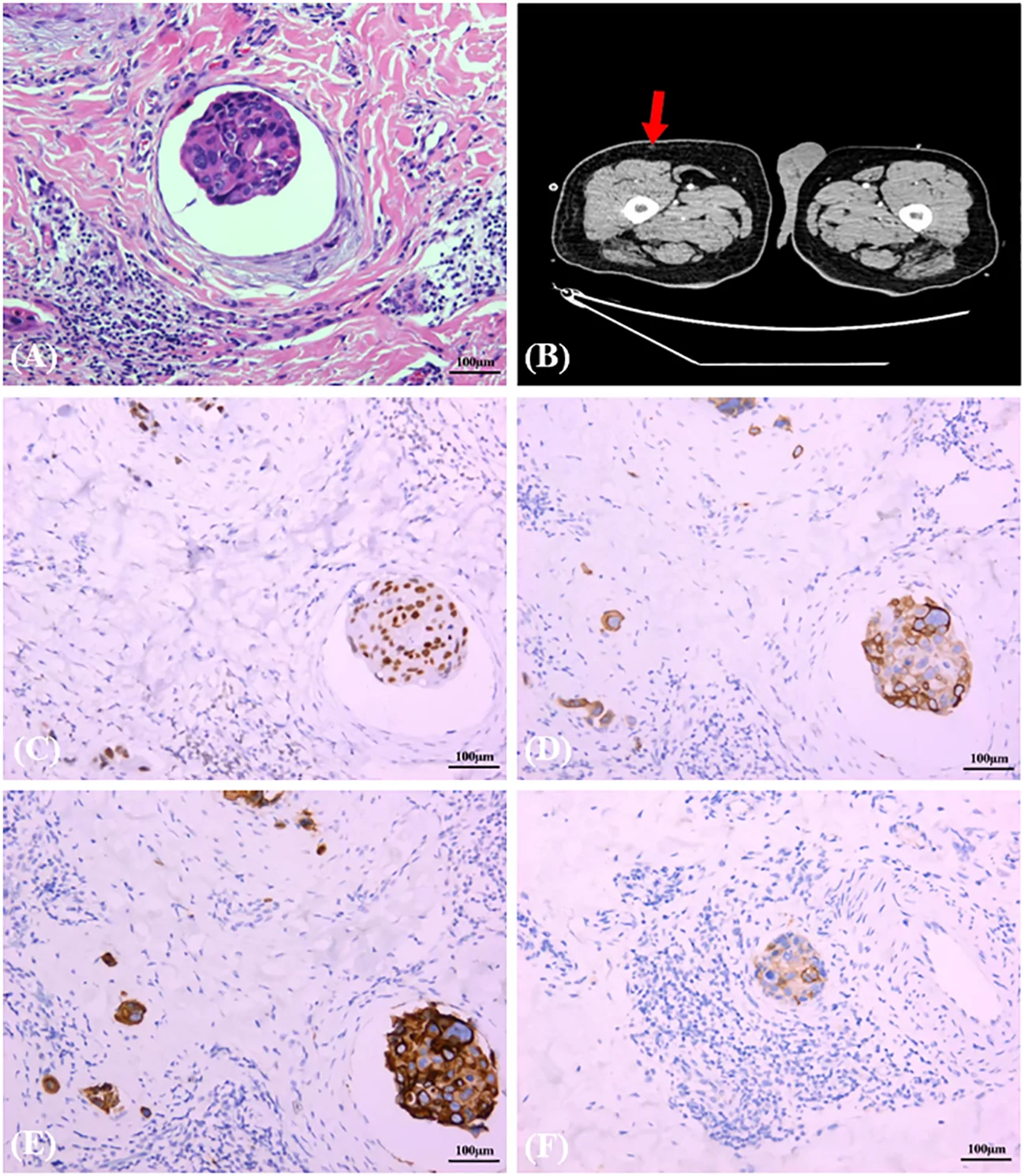
Histopathological, radiological, and immunohistochemical confirmation of cutaneous metastasis. **(A)** H&E stain of the skin biopsy. **(B)** CT image showing a 6.0 mm × 4.3 mm subcutaneous nodule (arrow) within the affected area of the right thigh. **(C)** GATA-3 (+). **(D)** CK7 (+). **(E)** CK20 (+). **(F)** Uroplakin II (+).

**Figure 3:**
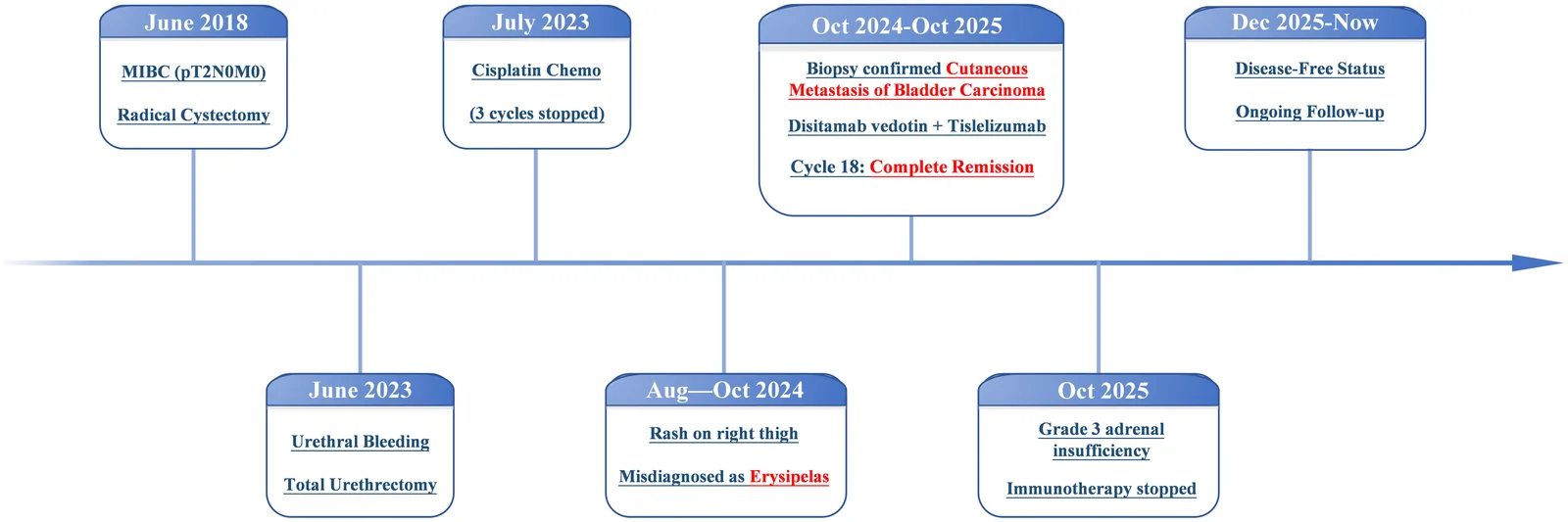
Timeline of disease course and treatment.

In August 2024, the patient noticed the development of a maculopapular rash on the right anteromedial thigh. He had no fever, chills, or other systemic symptoms suggestive of infection. Nevertheless, because of the local erythema, induration, swelling, and mild warmth, the lesion was initially treated as erysipelas in the Department of Dermatology with oral moxifloxacin for 2 months. No clinical improvement was observed. Instead, the lesion progressively enlarged and was accompanied by radiating pain and increasing leg swelling. The right thigh pain was rated as 6 on the Numeric Rating Scale (NRS). Physical examination revealed a 30 cm × 20 cm irregular and nonulcerated maculopapular rash with mild local warmth (**Figure 1**). The circumference of the right thigh was 60 cm, compared with 52 cm on the left side. In October 2024, the skin biopsy revealed malignant epithelial cells (**Figure 2A**). Immunohistochemistry was positive for GATA binding protein 3 (GATA-3), cytokeratin 7 (CK7), cytokeratin 20 (CK20), and Uroplakin II (partial positivity), supporting the diagnosis of metastatic urothelial carcinoma (**Figures 2C–F**). A further evaluation with computed tomography (CT) was then performed, which showed a newly developed 6.0 mm × 4.3 mm subcutaneous nodule within the affected area of the right thigh (**Figure 2B**). The disease course is illustrated in **Figure 3**.

**Figure 4:**
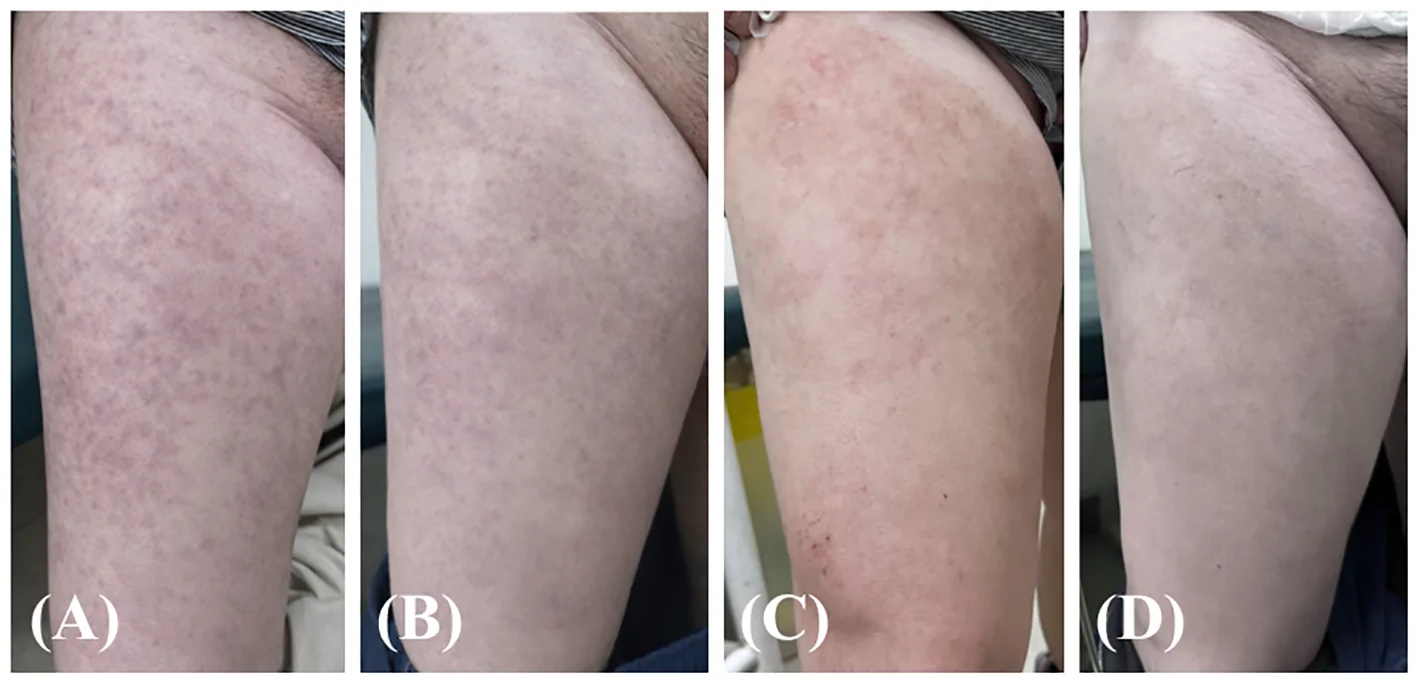
Sequential images demonstrating the therapeutic response to disitamab vedotin + tislelizumab treatment. **(A)** Cycle 4: mild regression compared with the initial presentation. **(B)** Cycle 9: partial resolution of swelling, with a noticeable reduction in lesion size. **(C)** Cycle 14: lesion visibly regressed, with softening of the local tissue. **(D)** Cycle 18: complete clinical resolution.

Based on his prior intolerance to cisplatin-based chemotherapy, tumor biomarker profile (HER2 2+, PD-L1 CPS = 20), and multidisciplinary assessment, combination therapy with disitamab vedotin (120 mg) and tislelizumab (200 mg) administered every 3 weeks was initiated in October 2024. Clinical improvement became evident during the treatment. By cycle 6, the patient’s pain had completely resolved (NRS 0). After 13 cycles, the circumference of the right thigh had decreased to 55 cm, and the skin lesion faded significantly. After 16 cycles, the lesion had nearly disappeared. Complete clinical and radiological remission was achieved by cycle 18, with the skin color restored to normal and the thigh circumference reduced to match that of the contralateral side. The progressive resolution of the skin lesion was documented in **Figure 4**. Surveillance imaging with abdominal and chest CT scans performed every 3 months demonstrated no evidence of disease recurrence at other sites. The patient adhered well to the treatment regimen and attended regular follow-up.

However, approximately 12 months after initiating immunotherapy, the patient developed symptoms of adrenal insufficiency, including nausea, anorexia, and weight loss. Laboratory evaluation confirmed the diagnosis, revealing profoundly low serum cortisol levels (8:00 AM: 15.37 nmol/L; 4:00 PM: 2.76 nmol/L) with inappropriately suppressed ACTH (8:00 AM: 1.1 pg/mL; 4:00 PM: 2.18 pg/mL). The adverse event was effectively managed with prednisone replacement therapy (initiated at 40 mg daily and tapered to a maintenance dose of 7.5 mg daily), leading to significant improvement in symptoms. Consequently, immunotherapy was permanently discontinued to avoid the risk of potentially life-threatening recurrence upon rechallenge. At the 2-month follow-up after discharge, the patient’s condition remained stable, with no recurrence of adrenal-related symptoms. At the latest follow-up, 14 months after treatment initiation, the patient remained free of tumor recurrence and continued active long-term surveillance.

## 3 Discussion

Cutaneous metastasis from bladder cancer is an uncommon but clinically important event, often associated with advanced disease and poor clinical outcomes (10). Skin biopsy with immunohistochemistry (IHC) is essential for establishing the diagnosis. CK7, CK20, and GATA3 are commonly expressed in urothelial carcinoma and may help distinguish metastatic urothelial carcinoma from other cutaneous malignancies (11, 12). In our patient, tumor cells were positive for CK7 and GATA3, with partial Uroplakin II expression, further supporting the diagnosis of metastatic urothelial carcinoma, as previously reported (11).

We reviewed 57 cases of cutaneous metastasis from bladder cancer reported in PubMed between 1 January 1949 and 1 February 2026 (**Supplementary Table 1**) (5–57). Among these, about 40% were associated with the abdominal wall or inguinal region, whereas only eight cases (14.0%) involved the thigh. Among the eight reported cases involving the thigh, none presented as an isolated cutaneous metastasis in the absence of concurrent extracutaneous metastases. Therefore, isolated thigh involvement without visceral metastasis appears to be particularly uncommon. Cutaneous metastases may clinically mimic benign dermatologic conditions. In our review, 49% presented as indurated erythematous nodules, and 18% resembled cellulitis- or erysipelas-like plaques. Initial misdiagnoses included herpes zoster (10), cellulitis (13), benign dermatologic conditions (14), or insect bites (15). In our patient, the lesion was initially misdiagnosed as erysipelas because of its erythematous and indurated appearance on the thigh. The lack of response to prolonged antibiotic therapy, together with progressive swelling and neuropathic pain, prompted skin biopsy and ultimately led to the correct diagnosis. These findings suggest that persistent inflammatory-appearing skin lesions in patients with a history of urothelial carcinoma should raise suspicion for cutaneous metastasis (11, 16–18).

The pathophysiology of cutaneous metastasis from bladder cancer has not been well studied and may vary by site. Lymphatic spread is considered an important route, particularly in patients with prior pelvic surgery. In our patient, a history of radical cystectomy and pelvic lymph node dissection may have altered normal lymphatic drainage and facilitated retrograde lymphatic dissemination of tumor cells. This may have resulted in carcinomatous lymphangitis, with diffuse erythema and brawny edema of the thigh (11, 16). Hematogenous dissemination may explain lesions that develop outside expected lymphatic pathways (17, 18). Although direct implantation during surgical manipulation has been reported as a possible mechanism, it seems less likely in the present case because the lesion was distant from the ureterocutaneous stoma. The unusual anatomical location may also have contributed to delayed recognition. Atypical distribution can lower clinical suspicion and increase the risk of misdiagnosis. In fact, several studies have described initial misidentification as infectious conditions such as cellulitis or erysipelas, often with no response to antibiotics, thereby delaying biopsy and definitive diagnosis (13, 16, 18).

Treatment options for cutaneous bladder cancer metastases should be considered within the broader context of the rapidly evolving systemic treatment landscape of advanced urothelial carcinoma. Although platinum-based chemotherapy was historically a major systemic treatment option, recent evidence has established an important role for ADC plus ICI strategies. EV-302/KEYNOTE-A39 demonstrated superior outcomes with enfortumab vedotin plus pembrolizumab compared with platinum-based chemotherapy in previously untreated locally advanced or metastatic urothelial carcinoma (4). More directly relevant to the present case, RC48-C016 showed that disitamab vedotin plus toripalimab significantly improved outcomes compared with chemotherapy in untreated HER2-expressing locally advanced or metastatic urothelial carcinoma (58). In this case, treatment selection was based on prior intolerance to cisplatin-based chemotherapy, HER2 expression (2+), high PD-L1 CPS, and multidisciplinary assessment. Although tislelizumab rather than toripalimab was used in our patient, both agents target PD-1. Therefore, the complete clinical and radiological remission observed after disitamab vedotin plus tislelizumab is consistent with the emerging rationale for combining HER2-directed ADC therapy with PD-1 blockade in appropriately selected patients with metastatic urothelial carcinoma.

However, treatment-related toxicity requires careful monitoring. Our patient developed grade 3 adrenal insufficiency, an immune-related adverse event (irAE), and was successfully treated with hormone replacement therapy. In this case, immunotherapy was permanently discontinued to avoid the risk of potentially life-threatening recurrence upon rechallenge. This case underscores the importance of maintaining a high index of suspicion for endocrine irAEs, as their initial manifestations are often nonspecific and require prompt endocrine evaluation, as recommended in the latest guidelines (1, 59).

The main strength of this report is the detailed clinicopathological, radiological, and therapeutic documentation of an isolated erysipelas-like cutaneous metastasis from urothelial carcinoma, including its response to biomarker-guided HER2-directed ADC plus PD-1 inhibitor therapy. Nevertheless, this is a single case report, and the efficacy of this treatment strategy in similar cutaneous metastatic presentations cannot be generalized. Further clinical evidence is needed to clarify the role of HER2-directed ADC plus PD-1 inhibitor therapy in this setting.

In conclusion, this case underscores the need to consider cutaneous metastasis in patients with bladder cancer, particularly when presumed infection does not respond to antimicrobial therapy. Early biopsy is required for diagnosis and may guide biomarker-directed therapy. Close collaboration between urologists, dermatologists, oncologists, and pathologists is important to ensure timely and appropriate management of these patients.

## 4 Patient perspective

The patient reported significant relief of pain and improvement in daily activities after treatment. He expressed satisfaction with the therapeutic outcome and agreed to continue regular follow-up and long-term surveillance.

## Data Availability

The original contributions presented in the study are included in the article/**Supplementary Material**. Further inquiries can be directed to the corresponding authors.
