# Supplementary material for: Case Report: Solitary right thigh skin metastasis of bladder cancer misdiagnosed as erysipelas with literature review
Source: Front Oncol. 2026 Sep 3;16:1825362. doi: 10.3389/fonc.2026.1825362 (PMC13581776; doi:10.3389/fonc.2026.1825362)
Supplement: Supplementary file 1 [file Table1.docx]

**Supplementary Table 1.** Reported cases of cutaneous metastasis originating from bladder cancer (1949-2026)

| **Author** | **Year** | **Sex** | **Age** | **Primary Surgery ± Adjuvant Therapy** | **Time to Skin Metastasis** | **Site of Skin Metastasis** | **Morphological Features** | **Initial Clinical Diagnosis/Misdiagnosis** | **Treatment of Cutaneous Lesion** | **Survival after Metastasis** | **IHC Markers** |
| --- | --- | --- | --- | --- | --- | --- | --- | --- | --- | --- | --- |
| Mohammed^1^ | 2024 | M | 57 | RC + ileal conduit+CT (Paclitaxel) | 2 mo (after surgery) | Left cheek | Erythematous nodule | NR | Surgical excision+CT (Paclitaxel) | Deceased (10 mo) | NR |
| Baczynski^2^ | 2024 | M | 79 | TURBT; RC+IT (Pembrolizumab)+ADC (Enfortumab Vedotin) | During cycle 3 of IT+ADC | Right inguinal fold and thigh | Multiple erythematous nodules coalesce into an irregular, indurated plaque | Herpes zoster | RT | NR | CK20+, GATA3+, CK7- |
| Mammadova^3^ | 2024 | M | 67 | TURBT+CT (BCG induction and maintenance) | 6 yr (after initial diagnosis) | Entire right lower extremity | Multiple ulcerating fungating lesions | Spider bite | RT+IT (Cemiplimab) | Deceased (septic shock) | Pan-keratin+, p63+, CK5/6+, p16+, GATA3+, CK7- |
| Izzo^4^ | 2023 | F | 63 | TURBT; RC+ureterectomy+CT(Gemcitabine, Carboplatin) | 2 mo (after RC) | Lower third of the right leg | Multi-nodular lesion | NR | IT (Pembrolizumab) | Deceased (2 mo) | CK7+, CK20+, GATA3+ |
| Hayoune^5^ | 2023 | M | 69 | TURBT+NAC (Cisplatin/Gemcitabine); RC | 1 yr (after RC) | Posterior cervical region and right breast | Cutaneous lesions | NR | Alliative care | Deceased (several wk) | CK7+, GATA3+ |
| Öztürk^6^ | 2022 | F | 80 | TURBT; RC+urethrectomy | 2 mo (after RC) | Abdomen | Reddish, painful lumps | Erythema Nodosum appearance | IT (Pembrolizumab) | NR | CK7+, GATA3+ |
| Otsuka^7^ | 2021 | F | 77 | TURBT+CT (Gemcitabine, Cisplatin) | After 8 courses of CT | Lower abdomen | Skin rash | Rash (suspected chemotherapeutic side effect) | IT (Pembrolizumab) | Deceased (5 wk) | CK7+, CK20+, GATA3+ |
| Yajima^8^ | 2021 | M | 70 | TURBT; NAC (Cisplatin/Gemcitabine); RARC | 2 mo (after RC) | Left chest | Reddish mass | NR | IT (Pembrolizumab) | Survived (>5 mo) | CK7+, CK20+, GATA3+ |
| Mathew^9^ | 2021 | M | 76 | NR | Synchronous diagnosis | Upper abdomen, back, scalp, left orbit | Firm, non-tender skin nodules | NR | Palliative CT (cisplatin-based) | Deceased (3 mo) | Pan-keratin+, CK7+, CK20+, GATA3+ |
| Feldmann^10^ | 2020 | M | 78 | TURBT; Re-TURBT+complete lymph node dissection+CT(Gemcitabine, Cisplatin) | 5 mo (after CT completion) | Lower extremities, genital area, lower abdomen | Erysipelas-like and cobblestone appearance | Erysipelas | IT (Pembrolizumab) | Deceased (3 mo) | CK7+, CK20+, GATA3+ |
| Hasan^11^ | 2020 | F | 71 | TURBT (Incomplete resection) | Present at initial diagnosis (metastatic disease) | Lower abdominal wall | Nodular and erythematous skin lesions | NR | CT (Cisplatin, Gemcitabine)+IT (Atezolizumab) | Deceased (1 wk after treatment) | p63+, p40+, CK7+, CK20+, uroplakin+ |
| Bhakta^12^ | 2020 | M | 71 | RC | 5 mo (after RC) | Left axilla | Vascular appearing, painless, and tethered on a stalk to the axillary fossa | NR | Surgical excision | NR | NR |
| Ghalleb^13^ | 2019 | M | 61 | RC+left ureteronephrectomy+lymph node dissection | 1 yr (after surgery) | Left hypochondriac, back, cervical region | Multi-nodular lesion | NR | NR | Deceased (1 mo) | CK7+, p63+ |
| Garg^14^ | 2018 | M | 62 | NR | Synchronous diagnosis | Neck, axilla, scapular region | Multiple subcutaneous swellings | NR | NR | Deceased (2 wk) | NR |
| Kerkeni^15^ | 2017 | M | 74 | RC+CT(Cisplatin-based) | 4 mo (after surgery) | Chest | Multiple disseminated pigmented flat lesions | NR | NR | NR | p63+ |
| Grace^16^ | 2017 | M | 61 | RC | 3 yr (after RC) | Bilateral inguinal, suprapubic, proximal medial thighs | Erythematous papules and nodules | Herpes zoster, cellulitis, fungal infection, hidradenitis suppurativa | CT (unclear) | Deceased (1 mo) | CK7+, CK20+ |
| Giunchi^17^ | 2017 | M | 69 | CT (Gemcitabine) | 7 mo (after initial diagnosis) | Foreskin, glans | Several brownish warty fragments | Condylomata acuminata | Laser therapy | Survived (>8 mo) | GATA3+, CK7+, CK20+, CK14(focal)+, p16- |
|  | 2017 | M | 71 | RC | 10 yr (after initial diagnosis) | Glans | Reddish skin lesion | NR | Complete glans resection | Survived (till last follow up) | GATA3+, CK7+ |
| Cohen^18^ | 2017 | M | 75 | TURBT; Re-TURBT+CT (intravesical BCG) | 8 mo (after initial diagnosis) | Right inguinal region, scrotum | Multiple nodular, fibrotic, hemorrhagic lesions | NR | NR | Deceased (shortly after diagnosis) | Pan-keratin+, CK7+, CK20-, p63- |
| Filho^19^ | 2016 | M | 63 | TURBT; Cystectomy | 1 mo (after RC) | Hypogastrium, suprapubic region | Along with surgical scar | NR | NR | Deceased (1 mo) | Vimentin+, cytokeratin AE1/AE3 (rare cells)+ |
| Lees^20^ | 2015 | M | 81 | RC | 8 yr (after RC) | Left anterior thigh | Macular nodular rash | Leg swelling and rash | Palliative RT | Deceased (6 mo) | CK7+, CK20+, CK5/6 (focal)+, p63 (focal)+, PSA-, TTF-1- |
| Xu^21^ | 2015 | M | 53 | PC+urachal remnant resection | 5 yr (after initial surgery) | Penis, scrotum, and groin | Erysipeloid-like mass | Benign skin lesion | Total resection of penis, scrotum, groin skin | Survived (>7 mo) | CAM5.2+, CDX2+, CA19-9+, p63- |
| Mitsui^22^ | 2014 | M | 48 | RC+CT (Gemcitabine, Cisplatin) | 17 mo (after RC) | Head and lower abdomen, choroid of the eye | Multiple erythematous nodules | Benign skin nodules | CT (Methotrexate, Vinblastine, Doxorubicin, Carboplatin) | Deceased (5 mo) | NR |
| Narayana^23^ | 2014 | M | 51 | TURBT | 10 mo (after TURBT) | Left subscapular region, umbilical region, right shoulder | Multiple nodular swellings | Multiple nodular swellings | NR | NR | NR |
| Agarwal^24^ | 2014 | M | 49 | TURBT+flank mass resection+CT (Gemcitabine, Cisplatin, Carboplatin) | Synchronous diagnosis | Right flank | Large subcutaneous mass | Large flank mass | Resection of flank mass+CT (Gemcitabine, Cisplatin and Carboplatin) | Deceased (17 wk) | CK7+, CK20+, p63+, AE1/AE3+; CK5/6-, PLAP-, napsin-, TTF-1-, CD117-, CDX2- |
| Chang^25^ | 2013 | M | 72 | CT (Gemcitabine) | Synchronous diagnosis | Suprapubic, chest, abdomen, prepuce, scrotum | Erysipelas-like plaques, later ulcerative | Right leg and suprapubic lymphedema; skin lesions ignored initially | NR | Deceased (6 mo) | CK7+, CK20+ |
| Chung^26^ | 2013 | M | 60 | RC; CT (Gemcitabine, Cisplatin) for recurrence | 10 yr (after RC) | Abdominal skin | Multiple erythematous nodules | Multiple abdominal skin nodules | NR | Deceased (the day after excisional biopsy from ARDS) | NR |
| Salmanpoor^27^ | 2013 | M | 33 | NR | Synchronous diagnosis | Whole back and posterior neck | Erythematous violaceous indurated plaque with illdefined borders and woody induration | Rapidly enlarging skin tumor | NR | NR | AE1/AE3+, CK7+, CK20+, EMA+, vimentin+ |
| Yoshida^28^ | 2012 | F | 65 | RC+pelvic lymphadenectomy + right nephroureterectomy | 4.5 yr (after primary surgery) | Adjacent to the left ureterocutaneostomy stoma | Painless skin erosion | Benign skin erosion | Tumor extirpation with stoma reconstruction + CT (Gemcitabine, Cisplatin) | Survived (>3 mo) | NR |
| Dirican^29^ | 2012 | M | 60 | RC | 1 mo (after RC) | Suprapubic region | Erythematous papular lesions, erysipeloid appearance | Cellulitis (treated with antibiotics) | CT (Carboplatin, Gemcitabine) | NR | CK7+, CK20+ |
| Manzelli^30^ | 2011 | M | 68 | TURBT | Synchronous diagnosis | Right chest wall | Hypodermic nodule | Gross haematuria | Surgical resection+CT (Gemcitabine, Carboplatin, Paclitaxel) | Survived (>6 mo with local progression) | CK7+, HER2+, CK20-, CD117-, TTF-1-; desmin+ in sarcomatoid component |
| Blalock^31^ | 2011 | M | 80 | TURBT+CT (intravesical BCG) | 18 mo (after initial diagnosis) | Left lower abdomen | Firm pink plaque with “frog spawn” appearance | Lymphangioma circumscriptum, atypical infection, lymphoma cutis, metastasis | CT (Carboplatin, Gemcitabine) | NR | Pan-keratin+, CK7+, CD20+, CK19+, CD138+ |
| Salemis^32^ | 2011 | M | 68 | RC+bilateral ureterostomy (17 months prior) | 17 mo (after RC) | Left lower abdomen | Palpable nodule | Palpable nodule | NR | Deceased (2 mo) | NR |
| Swick^33^ | 2010 | M | 69 | TURBT+CT (intravesical BCG) | 3 mo (after TURBT) | Mid-upper back and left mid-back | Violaceous-to-pink nodules | Asymptomatic skin lesion | NR | Deceased (several wk) | Pan-keratin+, CK7+, CK20+ |
| Sgambato^34^ | 2009 | F | 72 | Surgery+local CT (both unspecified) | Not specified (patient had a 7-year history of disease) | Scalp and back | Red, firm, nodular | Skin nodules | NR | NR | NR |
| Atmaca^35^ | 2007 | M | 66 | TURBT; CT (intravesical mitomycin); Re-TURBT | 4 mo (after initial diagnosis) | Trunk and upper extremities | Multiple subcutaneous metastatic nodules | Multiple subcutaneous nodules | CT (Gemcitabine, Carboplatin) | Deceased (10 mo) | NR |
| Kumar P^36^ | 2006 | M | 55 | NAC (gemcitabine, cisplatin) | 9 wk (after initial diagnosis) | Right arm | Firm, nontender, nonadherent nodule | Skin nodule with central crusting | NR | NR | NR |
| Somani^37^ | 2006 | F | 83 | TURBT | 6 wk (after TURBT) | Left side of neck and chest | Multiple reddish nodules | Herpetiform rash | Palliative RT | Deceased (6 mo) | CK7+, CK20+ |
| Zwenzner^38^ | 2006 | M | 68 | RC+lymphadenectomy+CT (Gemcitabine, Cisplatin, Paclitaxel, Carboplatin) | 3 yr (after RC) | Neck | Erythematous induration | Benign skin lesion | NR | Deceased (several weeks) | NR |
| Gowardhan^39^ | 2004 | M | 51 | TURBT; RT+RC | 3 mo (after RC) | Left iliac fossa skin | Singular nodule | NR | CT (oral methotrexate) | Survived (>23 yr) | NR |
| Akman^40^ | 2002 | M | 78 | TURBT+CT (Gemcitabine, Carboplatin) | 3 mo (after TURBT) | Trunk | Multiple, rubbery subcutaneous nodules | Benign skin lesion | CT (Gemcitabine, Carboplatin) | Deceased (10 mo) | NR |
| Miyamoto^41^ | 2000 | M | 75 | CT (Cisplatin, Doxorubicin) | 3 yr (after initial diagnosis) | Glans penis, corona glandis | Reddish oval tumor with a well-demarcated surrounding erythema and slight erosion | Inflammatory lesion | Topical CT (5-Fluorouracil) +corticosteroid ointment+Local resection after progress | Survived (>4 mo) | CK7+, CK20+, GATA3+ |
| KumarPV^42^ | 2000 | M | 58 | RT+CT (unspecified) | 7 mo (after initial diagnosis) | Right lumbar paraspinal region | Painful, soft and nonmobile subcutaneous mass | Abscess | RT | Deceased (10 mo) | NR |
| Saito^43^ | 1998 | M | 79 | TURBT+BCG infiltration | 18 mo (after TURBT) | Scrotum | Solitary subcutaneous induration | Benign skin lesion | Surgical excision+CT (MVAC) | Deceased (15 mo) | NR |
| Stolla^44^ | 1994 | M | 58 | Lymphadenectomy+CT (MVAC)+RT | 9 mo (after CT+RT) | Coelioscopy puncture scars | NR | Abscess | RT | Deceased (1 mo) | NR |
| Roos^45^ | 1993 | M | 75 | RT | 5 mo (after initial diagnosis) | Left neck | Several supraclavicular nodes | Inflammatory skin lesion | CT (Cisplatin)+RT | Deceased (8 mo) | NR |
| Fujita^46^ | 1994 | M | 72 | TURBT; RC+nephroureterectomy + lymph node dissection; CT (cisplatin, doxorubicin, cyclophosphamide)+RT | 2 mo (after RC) | Thighs, lower abdomen | Multiple erythematous eruptions | Multiple erythematous skin eruptions | Topical CT (Bleomycin ointment)+palliative care | Deceased (4 mo) | NR |
|  | 1994 | M | 77 | TURBT+CT (oral tegafur) | 3 mo (after TURBT) | Thighs, lower abdomen, cheek | Erysipeloid rash | Erysipeloid skin rash | CT (oral tegafur) | Deceased (6 mo) | NR |
| Wyldes^47^ | 1988 | M | 43 | RT | 3 mo (after RT) | Right forearm | Firm, tender, well-demarcated nodule with induration and erythema | Simple skin lesion | RT | Survived (>4 mo) | NR |
| Takematsu^48^ | 1984 | F | 60 | NR | 4 yr (after initial symptoms) | Right inguinal region to mid-thigh | Large papillomatous, cauliflower-like tumor | Squamous cell carcinoma | CT (Doxorubicin, Fluorouracil)+palliative care | Deceased (4 mo) | NR |
| Bachrach^49^ | 1973 | M | 77 | NR | 1.5 mo (after initial diagnosis) | Penis, neck, abdominal wall | Ulcerated lesions | NR | NR | Deceased | NR |
| Wormer^50^ | 1956 | M | 88 | TURBT+Intravesical radon seeds | 1 yr (before initial diagnosis) | Cheek, periorbital tissue | Plum-colored, boggy, cystic mass with edema and serosanguinous drainage | Cystic mass | Excision of cutaneous nodule+RT | Deceased (5 mo) | NR |
| Hamer^51^ | 1951 | M | 71 | Cutaneous ureterostomies only | Synchronous diagnosis | Left anterior abdominal wall | Subcutaneous nodule | NR | Excision of the nodule | Deceased (5 mo) | NR |
| Higgins^52^ | 1949 | M | 51 | TURBT+radon seed implantation | 21 mo (after initial diagnosis) | Abdomen, thorax, upper extremity | Multiple subcutaneous nodules | Cellulitis | Palliative care | Deceased (2.5 mo) | NR |
|  | 1949 | M | 52 | Bilateral ureterosigmoidostomy+RC | 15 mo (after RC) | Suprapubic incision, right biceps area, back of neck, chest wall | Subcutaneous nodules | Suture reaction | RT | Deceased (6 mo) | NR |
| Mccrea^53^ | 1949 | M | 73 | TURBT+RT+Fulguration | 2 yr (after TURBT) | Back and shoulders | Painful nodules | NR | NR | Deceased (1 mo) | NR |

**Abbreviations**: M, male; F, female; TURBT, transurethral resection of bladder tumor; RC, radical cystectomy; RARC, robot-assisted radical cystectomy; PC, partial cystectomy; NAC, neoadjuvant chemotherapy; AC, adjuvant chemotherapy; CT, chemotherapy; RT, radiotherapy; IT, immunotherapy; ADC, antibody-drug conjugate; ARDS, acute respiratory distress syndrome; BCG, Bacille Calmette-Guérin; MVAC, methotrexate, vinblastine, adriamycin, cisplatin; wk, weeks; mo, months; yr, years; Data presented as ">X months" indicate patients were alive at last follow-up; NR, not reported; CK, cytokeratin; CK7, cytokeratin 7; CK20, cytokeratin 20; CK5/6, cytokeratin 5/6; CK14, cytokeratin 14; CK19, cytokeratin 19; AE1/AE3, cytokeratin AE1/AE3; CAM5.2, cytokeratin CAM5.2; GATA3, GATA binding protein 3; p63, tumour protein p63; p40, deltaNp63 isoform; p16, cyclin-dependent kinase inhibitor 2A; EMA, epithelial membrane antigen; PSA, prostate-specific antigen; TTF-1, thyroid transcription factor 1; CDX2, caudal-type homeobox 2; CD117, cluster of differentiation 117; CD20, cluster of differentiation 20; CD138, cluster of differentiation 138; PLAP, placental alkaline phosphatase; CA19-9, carbohydrate antigen 19-9.

**References**

1	Mohammed MH, Mardnly F, Ghrer M *et al.* Unusual metastasis after radical cystectomy: case report. *J Surg Case Rep* 2024; **2024**: rjae112.

2	Baczynski A, Medhus E, Skopis P *et al.* Cutaneous metastasis of urothelial carcinoma. *IJU Case Rep* 2024; **7**: 5-7.

3	Mammadova J, Patil R, Herman T *et al.* Cutaneous Metastasis Following a Six-Year History of Bladder Urothelial Carcinoma in Situ: A Case Report. *Cureus* 2024; **16**: e61193.

4	Izzo P, Izzo L, Lai S *et al.* Nodular cutaneous metastasis of the leg in advanced urothelial bladder carcinoma: a case report and systematic literature review. *Front Oncol* 2023; **13**: 1216725.

5	Hayoune Z, Ramdani M, Tahri Y *et al.* Late Cutaneous Metastases of Bladder Urothelial Carcinoma: A Case Report. *Cureus* 2023; **15**: e38038.

6	Ozturk H, Yurtsever S, Ozer A *et al.* Cutaneous metastasis of bladder urothelial carcinoma; A rare conditions. *Urol Case Rep* 2022; **41**: 101955.

7	Otsuka I, Ueno T, Terada N *et al.* Cutaneous metastasis emerging during chemotherapy and progressing during immunotherapy for urothelial carcinoma. *IJU Case Rep* 2021; **4**: 363-6.

8	Yajima S, Nakanishi Y, Wantanabe R *et al.* Urothelial carcinoma of the bladder with cutaneous metastases after robot-assisted radical cystectomy. Case report. *Urol Case Rep* 2021; **38**: 101709.

9	Mathew JE, Sharma AP, Chatterjee D *et al.* Skin nodules and proptosis in a case of carcinoma urinary bladder: An unusual presentation. *Indian J Urol* 2021; **37**: 286-7.

10	Feldmann R, Wruhs M, Stella A *et al.* Carcinoma erysipelatoides from urinary bladder carcinoma. *Eur J Dermatol* 2020; **30**: 438-9.

11	Hasan O, Houlihan M, Wymer K *et al.* Cutaneous metastasis of bladder urothelial carcinoma. *Urol Case Rep* 2020; **28**: 101066.

12	Bhakta PS, Harris EA, Connolly MM. A patient with history of bladder cancer presenting with an axillary mass: A rare case of supradiaphragmatic cutaneous bladder cancer metastasis. *Urol Case Rep* 2020; **31**: 101157.

13	Ghalleb M, Ayadi MA, Slim S *et al.* Multiple cutaneous metastasis of synchronous urothelial carcinoma of the bladder and the renal pelvis: a case report. *J Med Case Rep* 2019; **13**: 34.

14	Garg G, Sharma A, Sharma A *et al.* Metastatic adenocarcinoma of bladder presenting with skin and visceral metastasis. *BMJ Case Rep* 2018; **11**.

15	Kerkeni W, Ayari Y, Charfi L *et al.* Transitional Bladder Cell Carcinoma Spreading to the Skin. *Urol Case Rep* 2017; **11**: 17-8.

16	Grace SA, Livingood MR, Boyd AS. Metastatic Urothelial Carcinoma Presenting as Carcinoma Erysipeloides. *J Cutan Pathol* 2017; **44**: 513-5.

17	Giunchi F, Vasuri F, Valerio V *et al.* Unusual asymptomatic presentation of bladder cancer metastatic to the penis. *Pathol Res Pract* 2017; **213**: 717-20.

18	Cohen T, Ricchiuti D, Memo M. Bladder cancer that metastasized to the skin: A unique presentation that signifies poor prognosis. *Rev Urol* 2017; **19**: 67-71.

19	Bernardes Filho F, Melo ASAd, Pires ARC *et al.* Cutaneous invasion from sarcomatoid urothelial carcinoma: clinical and dermatopathologic features. *Anais Brasileiros de Dermatologia* 2016; **91**: 73-9.

20	Lees AN. Cutaneous metastasis of transitional cell carcinoma of the urinary bladder eight years after the primary: a case report. *J Med Case Rep* 2015; **9**: 102.

21	Xu W-H, Yang Y-H, Zhu Y *et al.* Unusual presentation of urachal adenocarcinoma with genitourinary cutaneous metastasis. *Asian Journal of Andrology* 2015; **17**.

22	Mitsui Y, Arichi N, Inoue K *et al.* Choroidal and cutaneous metastasis from urothelial carcinoma of the bladder after radical cystectomy: a case report and literature review. *Case Rep Urol* 2014; **2014**: 491541.

23	Narayana MA, Patnayak R, Rukmangadha N *et al.* Cutaneous metastasis of transitional cell carcinoma of the urinary bladder: Cytological aspect. *J Cytol* 2014; **31**: 50-2.

24	Agarwal I, Bruney GF, Sands C *et al.* Cutaneous Metastases from Urothelial Carcinoma of the Bladder: A Rare Presentation and Literature Review. *West Indian Med J* 2014; **63**: 548-51.

25	Chang CPs, Lee Y, Shih HJ. Unusual presentation of cutaneous metastasis from bladder urothelial carcinoma. *Chin J Cancer Res* 2013; **25**: 362-5.

26	Chung JH, Lee JY, Pyo JY *et al.* Brain and skin metastasis from urothelial carcinoma of the bladder. *Korean J Urol* 2013; **54**: 66-8.

27	Salmanpoor R, Saki N, Sepaskhah M *et al.* A metastatic cancer to skin in an otherwise asymptomatic young man: an unusual presentation. *Dermatology Online Journal* 2013; **19**.

28	Yoshida T, Takayama H, Uemura M *et al.* Solitary Skin Metastasis Adjacent to Ureterocutaneostomy 4 Years After Radical Cystectomy for Bladder Cancer. *Japanese Journal of Clinical Oncology* 2012; **42**: 331-4.

29	Dirican A, Kucukzeybek Y, Somaliota I *et al.* Cutaneous and subcutaneous metastases from bladder carcinoma. *Contemp Oncol (Pozn)* 2012; **16**: 451-2.

30	Manzelli A, Quaresima S, Rossi P et al. Solitary skin metastasis from sarcomatoid carcinoma of the bladder: a case report. J Med Case Rep 2011; 5: 484.

31	Blalock TW, Haun PL, Lesher JL, Jr. Cutaneous metastases of transitional cell carcinoma clinically mimicking lymphatic malformation. *J Am Acad Dermatol* 2011; **65**: e112-e4.

32	Salemis NS, Gakis C, Zografidis A et al. Cutaneous metastasis of transitional cell bladder carcinoma: a rare presentation and literature review. J Cancer Res Ther 2011; 7: 217–19.

33	Swick BL, Gordon JRS. Superficially invasive transitional cell carcinoma of the bladder associated with distant cutaneous metastases. *Journal of Cutaneous Pathology* 2009; **37**: 1245-50.

34	Sgambato A, Buffon RB, Cota C et al. In vivo reflectance confocal microscopy for cutaneous metastasis of bladder adenocarcinoma. Arch Dermatol 2009; 145: 213–15.

35	Atmaca AF, Akbulut Z, Demirci A et al. Multiple subcutaneous nodular metastases from transitional cell carcinoma of the bladder. Pathol Oncol Res 2007; 13: 70–72.

36	Kumar P, Prasad N, Biswas G et al. Cutaneous metastasis from urologic tumors. Indian J Dermatol Venereol Leprol 2006; 72: 86.

37	Somani BK, Prita D, Grant S *et al.* Herpetiform cutaneous metastases from transitional cell carcinoma of the urinary bladder: immunohistochemical analysis. *J Clin Pathol* 2006; **59**: 1331-3.

38	Zwenzner EM, Kaatz M, Ziemer M. Skin metastasis of ‘nested type’ of urothelial carcinoma of the urinary bladder. J Cutan Pathol 2006; 33: 754–55.

39	Gowardhan B, Powles T, Oliver RTD et al. Twenty-three years of disease-free survival following cutaneous metastasis from a primary bladder transitional cell carcinoma. Int J Urol 2004; 11: 1031–32.

40	Akman Y, Cam K, Kavak A et al. Extensive cutaneous metastasis of transitional cell carcinoma of the bladder. Int J Urol 2003; 10: 103–04.

41	Miyamoto T, Ikehara A, Araki M et al. Cutaneous metastatic carcinoma of the penis: suspected metastasis implantation from a bladder tumor. J Urol 2000; 163: 1519.

42	Kumar PV, Salimi B, Musallaye A et al. Subcutaneous metastasis from transitional cell carcinoma of the bladder diagnosed by fine needle aspiration biopsy. A case report. Acta Cytol 2000; 44: 657–60.

43	Saito S. Solitary cutaneous metastasis of superficial bladder cancer. Urol Int 1998; 61: 126–27.

44	Stolla V, Rossi D, Bladou F et al. Subcutaneous metastases after coelioscopic lymphadenectomy for vesical urothelial carcinoma. Eur Urol 1994; 26: 342–43

45	Roos DE, Wigg DR. Skin infiltration within a radiotherapy portal for metastatic bladder cancer—case report and review of the clinical literature. Acta Oncol 1993; 32: 689–91.

46	Fujita K, Sakamoto Y, Fujime M et al. Two cases of inflammatory skin metastasis from transitional cell carcinoma of the urinary bladder. Urol Int 1994; 53: 114–16.

47	Wyldes MP, Osborn DE. Solitary cutaneous metastasis from transitional cell carcinoma of the bladder. Br J Urol 1988; 61: 164.

48	Takematsu H, Obata M, Sato Y et al. Skin metastasis of adenocarcinoma of the urinary bladder. Dermatologica 1984; 169: 363–64.

49	Bachrach P, Dahlen CP. Metastatic tumors to the penis. Urology 1973; 1: 359–62.

50	Wormer DC, Martin WJ, Emmett JL *et al.* Cutaneous Metastasis from a Carcinoma of the Bladder: Report of a Case. *Journal of Urology* 1956; **75**: 697-700.

51	Hamer HG, Nourse MH. Cutaneous metastasis from carcinoma of bladder. *J Urol* 1951; **65**: 850-2.

52	Higgins CC, Hausfeld KF. Cutaneous metastases from carcinoma of the urinary bladder; report of two cases. *J Urol* 1948; **59**: 879-86.

53	McCrea LE. Cutaneous Metastasis of Bladder Carcinoma. *Journal of Urology* 1949; **62**: 158-62.
